# Auditory Middle Latency Response in Children with and Without Dichotic Deficits

**DOI:** 10.3390/children13020189

**Published:** 2026-01-29

**Authors:** Samar Babaee, Deborah Moncrieff

**Affiliations:** 1 School of Communication Sciences and Disorders, University of Memphis, Memphis, TN 38152, USA; 2 Institute for Intelligent Systems, University of Memphis, Memphis, TN 38152, USA

**Keywords:** dichotic listening deficits, amblyaudia, dichotic dysaudia, auditory middle latency response, binaural integration, electrophysiology, auditory processing disorder, children

## Abstract

Background/Objectives: Amblyaudia (AMB) and dichotic dysaudia (DD) are distinct subtypes of dichotic listening deficits characterized by different behavioral profiles. AMB is associated with marked interaural asymmetry, whereas DD is defined by bilaterally poor but relatively symmetric performance. The present study investigated whether these behavioral distinctions are reflected in the auditory middle latency response (MLR). Specifically, we examined whether children with AMB exhibit asymmetric MLR patterns and whether children with DD demonstrate more symmetric responses, relative to typically performing (TYP) peers. Methods: Thirty-seven children aged 9–12 years with normal peripheral hearing were recruited through clinical referrals and community outreach. Participants were classified as AMB, DD, or TYP based on performance on standardized dichotic listening measures. MLRs were recorded in response to monaural click stimulation delivered to each ear at both slow and fast presentation rates. Peak-to-peak Na–Pa amplitude and latency were analyzed to assess ear- and electrode-related effects across groups. Results: Children with AMB showed significant ear effects, with larger Na–Pa amplitudes elicited by left-ear stimulation, particularly at electrode C4, consistent with their behavioral asymmetry. In contrast, the DD group exhibited minimal amplitude asymmetry but showed prolonged Na–Pa latencies for right-ear stimulation at faster presentation rates. TYP children demonstrated small, expected asymmetries without significant latency delays. No reliable electrode effects were observed across groups. Conclusions: The MLR differentiated between subtypes of dichotic listening deficits in ways that paralleled behavioral performance, with amplitude asymmetry characterizing AMB and rate-dependent latency differences observed in DD. These findings suggest that the MLR may provide complementary, objective information relevant to the characterization of distinct dichotic listening profiles in children.

## 1. Introduction

Dichotic listening refers to the ability to process different auditory signals presented simultaneously to both ears, a crucial skill for navigating complex acoustic environments. Dichotic listening tests assess binaural integration, which involves processing inputs from both ears, and binaural separation, which reflects the ability to focus on one auditory input while ignoring the other [1]. These tests are commonly included in clinical batteries to evaluate dichotic listening performance; however, poor performance on these tasks alone is not sufficient to diagnose dichotic deficits, and must be interpreted in conjunction with other behavioral and clinical findings [2,3]. Because these tests provide separate scores for each ear and reveal interaural asymmetry, they offer a unique window into lateralized auditory processing, making them especially useful in measuring how environmental signals are encoded in everyday listening situations [4]. Based on ear-specific patterns, four functionally distinct profiles of dichotic listening performance have been identified [3,5]. Children performing at age-appropriate levels in both ears are identified as typical (TYP) [1]. Amblyaudia (AMB) is characterized by marked interaural asymmetry, in which the dominant ear performs at an age-appropriate level while the nondominant ear shows substantially poorer performance [5]. Dichotic dysaudia (DD), in contrast, is defined by bilaterally poor performance without substantial asymmetry. A fourth category, amblyaudia plus (AMB+), combines these two patterns, showing both low scores bilaterally and high asymmetry [3].

Kimura’s classic structural model of dichotic listening proposed that ear-specific deficits result from disrupted interhemispheric transfer, particularly when auditory input from one ear fails to cross the corpus callosum and reach the language-dominant hemisphere. This explanation emphasizes the role of interhemispheric transfer and has been supported by neuroanatomical studies in adults with corpus callosum lesions [6,7,8]. However, many children with dichotic listening deficits do not exhibit any identifiable structural lesions, yet they still demonstrate similar patterns of reduced performance on dichotic tasks [9,10].

The hypothesis guiding this study suggests that the source of the interaural asymmetry may be earlier in the auditory pathway, at the level of binaural integration within the brainstem. We propose that AMB reflects an imbalance in how excitatory and inhibitory signals are integrated within the superior olivary complex, where timing and intensity cues from both ears are processed to aid the listener’s ability to localize a relevant environmental stimulus [11]. As the first location in the auditory pathway where signals from the two ears converge, nuclei in the superior olivary complex balance ipsilateral excitation and contralateral inhibition to encode signals arriving from the two ears [12]. Excessive suppression of input from the nondominant ear by dominant crossover projections may lead to reduced neural activation along the ascending auditory pathway serving that ear, thereby producing pronounced interaural asymmetry [11,13]. While this subcortical disruption can originate in the brainstem, the neural consequences may propagate throughout multiple components of the ascending nervous system, leading to asymmetries that are also observable at the cortical level during evoked potential recordings.

Electrophysiological studies have reported delayed and/or reduced neural responses in some individuals diagnosed with APD, even in the absence of structural lesions. However, findings related to auditory evoked potential (AEP) measures have been inconsistent across studies and AEP components, likely reflecting variability in diagnostic criteria for APD, which range from performance-based auditory test batteries to multidisciplinary frameworks that incorporate language, attention, and cognitive measures. This variability contributes to substantial heterogeneity in participant characteristics [14,15,16,17,18,19,20,21]. Using various cortical evoked potential measures, Jirsa (1990) reported prolonged interpeak latencies and reduced response amplitudes in children identified with APD [14], with subsequent evidence of latency normalization and amplitude enhancement following auditory intervention (Jirsa, 1992), which indicates experience-dependent neural plasticity [15]. Similarly, Liasis et al. (2002) and Tomlin et al. (2016) reported altered latencies and reduced amplitudes in early cortical auditory evoked potentials in children diagnosed with APD, although effects varied across components and studies [16,17].

Within this broader AEP literature, Schochat et al. (2010) reported that children identified with APD exhibited significantly reduced amplitudes at the C3 electrode site for the auditory middle latency response (MLR) compared to controls. After the children received auditory training, MLR amplitudes significantly increased, particularly at C3-A1 and C3-A2 derivations, providing further evidence that subcortical and thalamocortical auditory responses can be modulated through intervention [18]. Mattsson et al. (2019) also investigated multiple AEP measures and found that the latency of the Na component of the MLR and the P300 event-related component were both sensitive to listening difficulties. Moderate correlations were observed between P300 latency and amplitude and behavioral auditory processing measures with competing words, frequency patterns, and dichotic digits, supporting the relevance of electrophysiologic markers for characterizing auditory dysfunction as revealed through behavioral assessments [19].

Within this context, electrophysiological studies focusing specifically on dichotic listening profiles have begun to reveal neural signatures that align with behavioral asymmetries across ears. A recent electrophysiologic study in children with AMB revealed stronger phase-locked oscillatory responses within the bilateral auditory cortices during right-ear stimulation and concurrently weaker functional connectivity from the right to the left auditory cortex [20], a neural asymmetry that aligned with their behavioral ear advantage profiles. These results were interpreted as evidence of a hyper-synchronization imbalance in sensory encoding and reduced interhemispheric integration in children with AMB. In parallel, functional imaging work has also shown asymmetric cortical activity during dichotic listening, with significantly heightened activation in the left hemisphere of children with AMB compared to controls [21]. Together, these findings suggest that dichotic listening deficits in AMB are associated with both exaggerated unilateral neural responses and disrupted bilateral communication, reinforcing the relevance of evaluating interaural asymmetries at both the subcortical and cortical levels.

The MLR reflects thalamocortical neural activity occurring approximately 16–80 ms after stimulus onset and provides a useful index of auditory system integrity in both children and adults [22]. It has been widely applied to examine developmental maturation, hemispheric differences, and the effects of stimulus parameters such as rate and ear of presentation, as well as neural transmission across interhemispheric pathways including the corpus callosum [23,24,25]. A well-recognized limitation of the MLR is substantial inter-subject variability in the absolute amplitude and latency of the Na and Pa components [26,27], which can complicate interpretation of group differences, particularly in clinical and pediatric populations [28,29]. In children, maturational changes in thalamocortical pathways further contribute to this variability, even in typically developing listeners [30]. To reduce these effects, relative peak-to-peak (PP) Na–Pa amplitude and latency measures have been recommended, as they provide more stable and reliable indices of auditory function, particularly for assessing interaural or interhemispheric asymmetries [31].

One goal of MLR waveform analysis is to evaluate the symmetry of responses across ears or electrode sites. While absolute MLR measures can vary between individuals, intra-subject consistency is often preserved at scalp regions overlying the auditory cortex, particularly at electrodes C3 and C4 [32]. Reductions in response amplitude over one hemisphere, regardless of the stimulated ear, are referred to as the “electrode effect” and have been observed in patients with cortical lesions [26,33], especially those involving the temporal lobe. Conversely, lesions located subcortically [34,35], in the thalamus or upper brainstem, often manifest as an “ear effect”, characterized by a reduced response following stimulation of the contralateral ear only [33,36].

Despite the promise of MLR in identifying neural asymmetries, its application in populations without frank lesions, such as children with auditory processing difficulties, remains limited and somewhat inconclusive. Prior efforts to use MLR for differentiating such populations have yielded inconsistent results and have yet to establish clear identification criteria or response [18,31,37]. The present study aims to address this gap by applying MLR waveform analysis to well-characterized subgroups of children with distinct dichotic listening profiles, with the goal of identifying MLR characteristics consistent with their behavioral profiles.

A major limitation in previous efforts to characterize auditory dysfunction in populations without documented structural lesions using electrophysiological measures has been the considerable heterogeneity across participants, assessment protocols, and identification definitions. To address this issue, the present study classified participants into three well-defined dichotic listening groups (typical, amblyaudia, and dichotic dysaudia) and utilized relative measures of MLR amplitude and latency to minimize variability in electrophysiologic responses. Building on prior research in children with AMB that demonstrated asymmetries in auditory processing between ears [20] and between hemispheres [21], this study aims to determine whether such asymmetries are reflected in MLR waveforms, specifically through ear and/or electrode effects. We further examine whether children with amblyaudia or dichotic dysaudia exhibit distinct patterns of MLR responses, which may correspond to their differing behavioral profiles.

## 2. Materials and Methods

### 2.1. Participants

The study sample consisted of 42 native English-speaking children aged 9 to 12 years (mean age = 10.8 years) with no reported history of neurological disorders, head trauma, or chronic medical conditions. All participants demonstrated normal peripheral hearing sensitivity on audiometric screening. Children were recruited either through community outreach via flyers or through clinical referrals for auditory processing evaluations. Clinical referrals were prompted by concerns regarding functional listening and academic or school-related performance, typically reported by parents or teachers, despite normal hearing acuity and the absence of documented peripheral hearing loss. Informed consent from a parent and child assent were obtained for all participants in accordance with procedures approved by the institutional review board overseeing this study.

### 2.2. Behavioral Evaluations

Pure-tone air-conduction thresholds were obtained for each ear using calibrated two-channel clinical audiometers (Grason-Stadler Model 16 or Maico Model 52). Thresholds were measured at octave frequencies from 1000 to 4000 Hz using TDH-45 supra-aural headphones, following a standard modified Hughson–Westlake procedure. Thresholds were defined as the lowest intensity level (in dB HL) at which the participant responded reliably to the stimulus on at least 50% of presentations.

Each participant completed two or three dichotic listening tests. The tests administered included the Dichotic Words Test (DWT), the Randomized Dichotic Digits Test (RDDT), and the Competing Words subtest (CW) from Screening Test for Auditory Processing Disorders (SCAN) [38,39,40]. Each test was administered according to standard instructions at 50 dB HL. In the Randomized Dichotic Digit Test, single-syllable digits from 1–10 (except 7) were presented dichotically to the right and left ears [39]. The presentation consisted of 18 pairs each of single digits (*n* = 18 digits), double digits (*n* = 36 digits), and triple digits (*n* = 54 digits). The Dichotic Words Test presents duration-matched single-syllable words dichotically. All stimuli were presented via headphones through a clinical audiometer set at 50 dB HL [38]. Participants were instructed to repeat the dichotic stimuli presented to both ears. Response order was not constrained for the RDDT or DWT, whereas specific reporting instructions were followed for the Competing Words subtest. They were instructed to make a guess if they were not sure what they heard. Individual scores in the right and left ears were obtained and converted to percent correct. For each participant, the ear yielding the higher percent correct score across tests was designated as the dominant ear, and the ear with the lower score was labeled the non-dominant ear. Interaural asymmetry was then calculated as the absolute difference between these two scores so that asymmetry values were always positive.

Individual ear scores were compared to cut-off values representing the 5th, 10th and 25th percentile of children within the same age group and were marked if the scores were not age appropriate. When the resulting score pattern for two tests matched, the results were characterized as either typical (TYP) or AMB or DD [1,3,5]. In some cases, poor scores were not consistent across the first two tests and the Competing Words subtest of the SCAN was used as a tiebreaker to establish group classification. This subtest consisted of 30 trials, presented in two sequential blocks of 15 trials each. In the first block, participants were instructed to repeat both words in each trial, beginning with the word heard in the right ear (“right ear first” condition). In the second block, the same procedure was followed with the instruction to begin with the left ear (“left ear first” condition). Each response was scored as either correct or incorrect, and the number of correct responses was summed separately for each ear, resulting in right and left ear scores out of 30. Interaural asymmetry was compared within each directed response condition to established normative prevalence rates of 5%, 10%, 15% or Typical [40].

Participants were categorized first on the direction of ear advantage, defined by which ear yielded higher percent correct scores on dichotic listening tasks, as demonstrating a right ear advantage (REA) or left ear advantage (LEA). The ear advantage is presumed to reflect hemispheric lateralization based on stronger activation along contralateral pathways following inputs of auditory signals to the opposite ear [41,42]. To effectively analyze the ear effect, we excluded the 5 children who demonstrated a LEA on both dichotic tests because their electrophysiologic results would also presumably be reversed. A second categorization was based on a comparison of ear scores and interaural asymmetries to age-appropriate cut-off values for each dichotic test. Participants were grouped into three categories beginning with TYP if all results were age-appropriate or AMB, DD, or AMB+ if their scores resulted in matched deficit patterns across both tests. Because the AMB+ pattern comprises the AMB and DD performance patterns, 2 children with the AMB+ pattern were placed into the DD group because their dominant ear was also unable to perform at typical levels. The final 37 REA participants who formed the primary sample for group-level statistical analyses included 17 children with AMB, 9 children with DD, and 11 typical controls.

### 2.3. Electrophysiology Assessment

All recordings were conducted in a sound-attenuated, electrically shielded booth. Electrophysiological data were collected using a 64-channel electrode cap (Quik-Cap; Neuroscan, Compumedics USA, Charlotte, NC, USA) secured with a chin strap, with electrode placement conforming to the international 10–20 system. Each electrode site was filled with a water-soluble conductive gel. EEG signals were recorded using a Neuroscan SynAmps2 amplifier (Compumedics USA, Charlotte, NC, USA) at a sampling rate of 10 kHz. Electrode impedances were measured prior to data acquisition and rechecked following completion of the recording to ensure values remained below 5 kΩ. All electrodes met the impedance criterion both before and after the recording session.

The stimulus used to elicit the MLR was a 385 μsec biphasic acoustic click, consisting of a 181 μsec first phase followed by a 204 μsec second phase. Clicks were delivered monaurally through ER-3A insert earphones at 60 dB nHL to each ear. Two stimulus rates were employed: a slow rate of 9.1 stimuli per second and a fast rate of 12.1 stimuli per second. Each participant completed four blocks of 1000 trials, resulting in approximately 6.5 min of active stimulus presentation and a total MLR session duration of approximately 7–8 min including brief inter-block pauses.

Evoked responses were recorded using silver/silver chloride (Ag/AgCl) electrodes embedded in the QuikCap, positioned according to the International 10–20 electrode placement system. Recording electrodes were referenced to linked mastoids, with the ground electrode placed at the mid-forehead location (AFz). Data were acquired at a sampling rate of 10,000 Hz and bandpass filtered online between 10 and 2000 Hz. Each recording epoch spanned from –10 to 65 milliseconds relative to stimulus onset, representing the maximum window permitted by the faster stimulus rate.

Following segmentation, epochs were subjected to baseline correction for the pre-stimulus interval, linear detrending for the entire window, and averaging. Artifact-free epochs were averaged within each condition to produce the final waveforms. A post-hoc low-pass filter at 750 Hz (12 dB/octave roll-off) was then applied to all averaged data to reduce high-frequency noise.

### 2.4. Analysis

To evaluate behavioral ear asymmetries, a one-way ANOVA was performed on ear advantage scores derived from the RDDT and DWT using IBM SPSS Statistics 30. The independent variable was the deficit group (TYP, AMB, DD), and the dependent variable was the ear interaural asymmetry. Following significant omnibus results, Tukey’s HSD post hoc tests were used to identify group differences in asymmetry.

To examine group differences and within-group effects on MLR peak-to-peak amplitude and latency, both descriptive and inferential statistical methods were employed. A series of univariate analyses of variance (ANOVAs) were conducted to assess the main effects and interactions of deficit group (TYP, AMB, DD), ear of stimulation (left, right), stimulus rate (slow, fast), and electrode site (C3, C4). The primary outcome variables were PP amplitude, defined as the voltage difference between the Na and Pa peaks, and PP latency, defined as the time interval between the most negative Na peak (14–25 ms) and the subsequent most positive Pa peak (21–45 ms). Following significant ANOVA results, Tukey’s HSD post hoc tests were performed to explore between-group differences. Additional within-group comparisons were conducted using stratified analyses at fixed levels of stimulus rate, ear, and electrode site to further explore patterns of asymmetry and rate sensitivity within each deficit group.

Two types of MLR response asymmetries were evaluated: ear effect, indicated by differences in response amplitude between left and right ear stimulation at a fixed electrode, and electrode effect, defined as differences in response amplitude between C3 and C4 at a fixed ear of stimulation. In cases where a significant ear or electrode effect was observed within groups, repeated-measures ANOVAs were conducted to determine whether the magnitude of these effects differed significantly between dichotic listening profiles.

## 3. Results

### 3.1. Behavioral Tests

**Figure 1:**
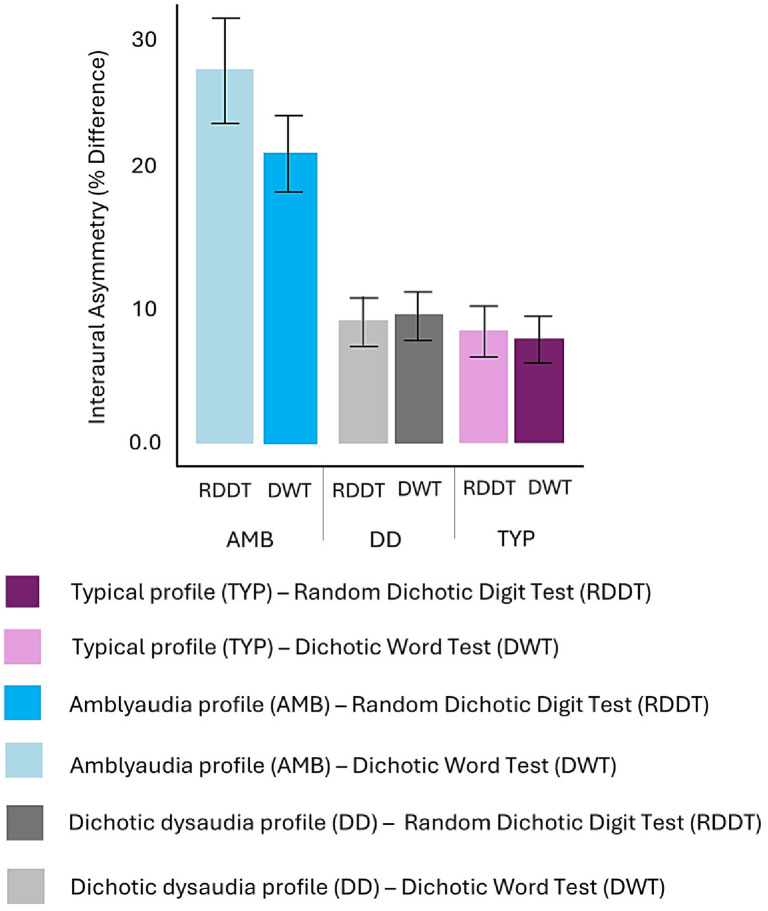
Average interaural asymmetry (% difference in correct responses) on the RDDT and DWT tasks. The AMB group showed greater interaural asymmetry compared to both the DD and WNL groups on both tasks. No significant differences were observed between the DD and WNL groups. Error bars represent standard errors.

Pure-tone audiometric thresholds were measured and all children demonstrated normal hearing sensitivity in both ears (≤25 dB HL at 500 Hz and ≤20 dB HL at 1000–4000 Hz). Average non-dominant and dominant ear scores within the three groups are reported in Table 1 and average ear advantage scores within the three groups are displayed in Figure 1. A one-way ANOVA on ear advantage scores revealed significant group differences for both, the RDDT F(2, 34) = 12.07, *p* = 0.0001, and the DWT F(2, 34) = 13.68, *p* < 0.001. Post hoc Tukey’s HSD tests showed that the AMB group exhibited significantly larger ear advantage scores than the TYP and DD groups on the RDDT (*p* = 0.001 and *p* < 0.001, respectively) and on the DWT (*p* < 0.001) for both groups. No significant differences were observed between the DD and TYP groups for either test.

### 3.2. MLR Findings

#### 3.2.1. Ear Effects on Peak-to-Peak Amplitude

**Figure 2:**
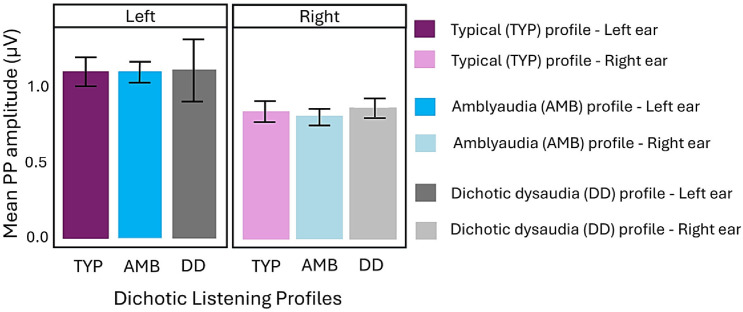
Mean (±SE) PP amplitudes (µV) for left- and right-ear stimulation in all three groups.

An analysis of variance (ANOVA) on PP amplitudes revealed a significant main effect of ear across all three groups, with larger amplitudes recorded during left-ear stimulation (M = 1.12 μV, SE = 0.07) compared to right-ear stimulation (M = 0.79 μV, SE = 0.03), F(1, 272) = 14.53, *p* < 0.001. To further explore ear-specific differences within each deficit group, the dataset was stratified by group, and PP amplitudes were compared between left and right ears using ANOVA with ear as the within-subject factor. As displayed in Figure 2, this within-group analysis consistently showed significantly larger amplitudes elicited by left-ear stimulation in both, AMB F(1, 156) = 10.11, *p* = 0.002 and TYP F(1, 78) = 5.76, *p* = 0.019 groups. The DD group also showed larger amplitudes for the left ear, but it did not reach a significant level due to high variability in the left ear response as shown in the error bars for that group.

To determine whether the magnitude of this ear effect differs significantly between the AMB and TYP groups, we conducted repeated-measures ANOVA with ear (left vs. right) as the within-subject factor and dichotic listening profiles (TYP vs. AMB) as the between-subject factor. The Ear × Group interaction was not significant, indicating that the magnitude of the PP amplitude for left and right ear stimulation was similar within each of these groups.

**Figure 3:**
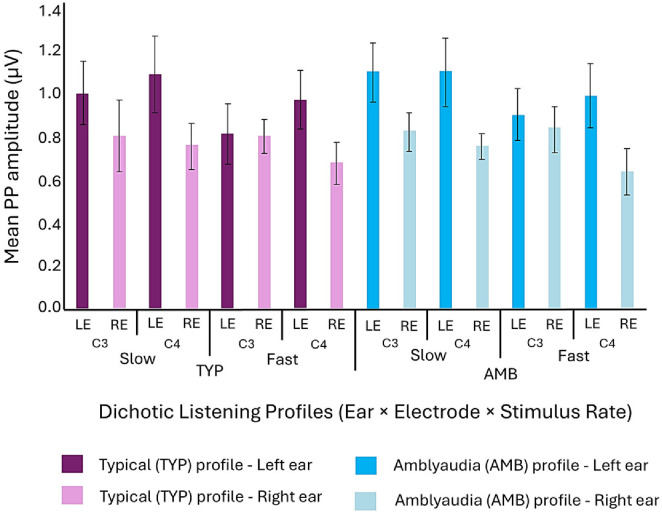
Mean (±SE) PP amplitudes (µV) in AMB and TYP groups at C3 and C4 electrode sites for left- and right-ear inputs.

The dataset for left versus right ear stimulation within the AMB and TYP groups was further stratified by rate (fast, slow) and electrode site (C3, C4) and an ANOVA was conducted with ear as the within-subject factor to determine if the larger left ear amplitude varied by rate of stimulation or by its measure at the scalp location over the right or left hemisphere. PP amplitude for left ear input was larger at electrode site C4 within both the AMB and TYP groups, illustrated in Figure 3.

**Figure 4:**
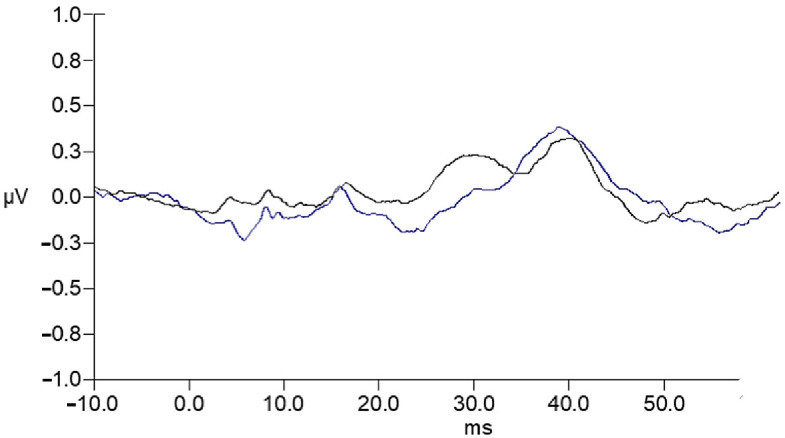
Grand-average waveforms illustrating PP amplitude in the AMB group for left-ear (blue) and right-ear (grey) stimulation, recorded at electrode site C4 during the slow stimulation rate condition. PP amplitude was significantly greater for left-ear stimulation compared to right-ear stimulation at this electrode and rate.

In both groups, larger PP amplitudes were evident at electrode site C4 for both fast and slow rates, but these differences achieved significance only for the AMB group at the fast rate F(1, 37) = 4.30, *p* = 0.045 and slow rate, F(1, 37) = 4.24, *p* = 0.047. The effect at the slow rate for the AMB group is shown in Figure 4. The larger PP amplitude observed at electrode site C4 is consistent with increased neural activity ascending through the presumptively dominant contralateral pathway to the scalp location over the right hemisphere following input of non-verbal stimulus to the left ear.

#### 3.2.2. Electrode Effects on Peak-to-Peak Amplitude

To evaluate electrode effects on PP amplitude, repeated-measures ANOVAs were conducted with electrode site (C3, C4) as the within-subject factor, stratified by deficit group, ear of stimulation, and stimulus rate. Across all conditions, no significant main effects of electrode site were observed (all *p* > 0.25), indicating that PP amplitudes did not differ systematically between C3 and C4.

#### 3.2.3. Stimulus Rate Sensitivity on Peak-to-Peak Amplitude

To examine how PP amplitude responds to different stimulus rates, we analyzed rate sensitivity within each group by comparing PP amplitudes elicited by fast and slow stimulation under fixed ear and electrode conditions. No significant main effects of stimulus rate were observed for any dichotic listening profile, with PP amplitude remaining comparable across fast and slow rates at all electrode sites and ears (all *p* > 0.38).

#### 3.2.4. Peak-to-Peak Amplitude in Children with LEA

**Figure 5:**
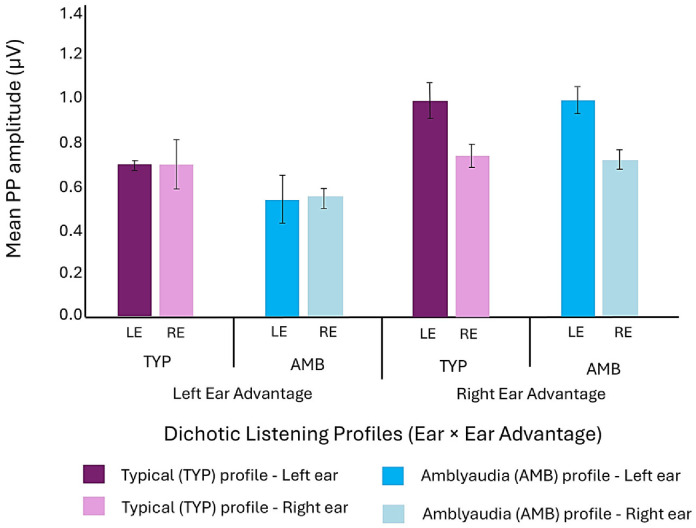
Average PP amplitudes (µV ± SE) for children with AMB and TYP across both right-ear advantage and left-ear advantage groups for left- and right-ear inputs.

Given that a larger amplitude of the MLR for left-ear input is typical for non-verbal stimuli in individuals who demonstrate a REA when performing a dichotic listening test with verbal stimuli, we evaluated the MLR responses from the children who were excluded from our prior analysis because they demonstrated a LEA when tested dichotically. Instead of demonstrating a similar reversal during the MLR and producing larger PP amplitudes following inputs to their right ears, the LEA children in the AMB and TYP groups showed highly symmetrical responses across ears (see Table 2). Interestingly, they also produced smaller PP amplitudes than the REA children in their groups. Figure 5 shows average PP amplitude across both REA and LEA groups for left and right ear inputs.

#### 3.2.5. Between-Group Differences in Peak-to-Peak Latency

The analysis of variance (ANOVA) for PP latency showed no significant main effects for deficit group, stimulus rate, or electrode site. However, several significant two-way interactions emerged, suggesting more complex interdependencies among the dichotic listening profiles, stimulus rates, and ear of stimulation. A significant Group × Rate interaction, F(2, 272) = 6.52, *p* = 0.002, revealed that stimulus rate had a significant effect on PP latency that varied across groups. As shown in Table 3, rate did not affect PP latency value for children in the TYP group. In contrast, PP latencies were longer with the slow rate in the AMB group and longer with the fast rate in the DD group.

A significant Rate × Ear interaction, F(1, 272) = 4.31, *p* = 0.039, indicated that latencies varied across ears and rates across all three groups. As shown in Table 4, longer PP latencies occurred for left ear stimulation at the slow rate whereas the opposite occurred with longer latencies for right ear stimulation at the fast rate. To further clarify these interaction effects, follow-up analyses were conducted by stratifying the data according to stimulus rate (slow, fast), ear of stimulation (left, right), and electrode site (C3, C4). Within each condition, PP latencies were compared between groups.

**Figure 6:**
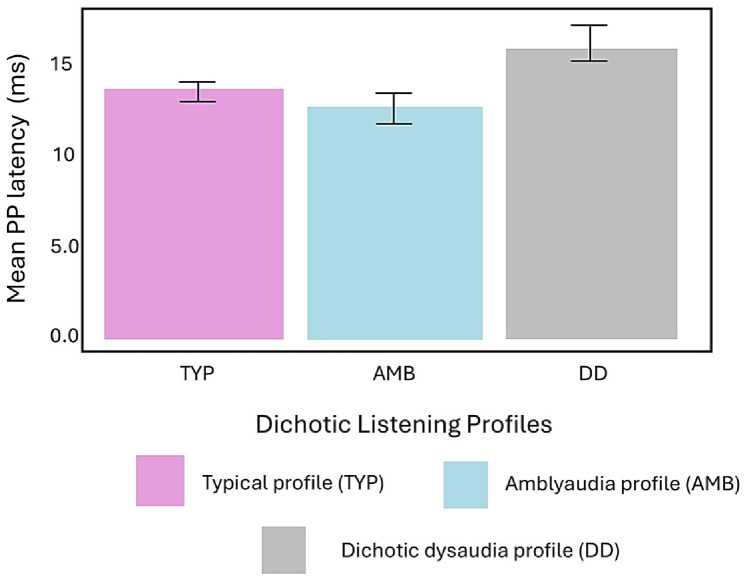
Mean (±SE) PP latency for all three groups. The DD group showed significantly longer PP latency compared to the AMB group.

Significant group differences emerged for fast-rate stimulation of the right ear at the contralateral electrode site C3, as shown in Figure 6, F(2, 34) = 3.62, *p* = 0.038. Post hoc Tukey analysis revealed that the DD group exhibited significantly longer PP latencies only compared to the AMB group (*p* = 0.029) that showed the shortest average latencies.

#### 3.2.6. Ear Effect on Peak-to-Peak Latencies

To further examine within-group ear effects, data were stratified by deficit group, stimulus rate, and electrode site, and PP latencies were compared between ears. In the DD group, a significant ear effect was observed at C4 during fast-rate stimulation, F(1, 12) = 5.09, *p* = 0.043. Specifically, right-ear stimulation elicited longer PP latencies compared to left-ear stimulation.

#### 3.2.7. Electrode Effect on Peak-to-Peak Latencies

To assess whether PP latency differed between electrode sites, we compared responses at C3 and C4 while holding stimulus rate and ear constant. This analysis was conducted using ANOVA with electrode site as a within-subject factor, applied to data stratified by deficit group, ear of stimulation, and stimulus rate. No significant electrode effects were observed for any dichotic listening profile, with PP latencies remaining comparable between C3 and C4 across all conditions (all *p* > 0.36).

#### 3.2.8. Stimulus Rate Sensitivity on Peak-to-Peak Latency

**Figure 7:**
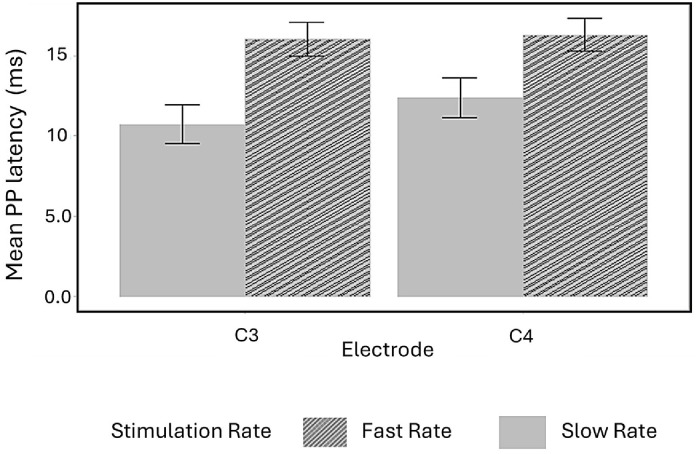
Within-group comparisons of peak-to-peak (PP) latencies across stimulation rates and electrode sites (C3 and C4) in the DD group. Fast-rate stimulation of the right ear elicited significantly longer latencies compared to slow-rate stimulation at both electrode sites. Error bars represent standard errors.

Lastly, to examine how PP latency responds to different stimulation rates, we analyzed rate sensitivity within each group by comparing responses to fast and slow stimulation under specific ear and electrode conditions. In the DD group, PP latency was significantly longer during fast-rate stimulation of the right ear compared to slow-rate stimulation at both electrode sites (C3: F(1, 12) = 11.28, *p* = 0.006; C4: F(1, 12) = 5.87, *p* = 0.031). Descriptive statistics are presented in Table 5, and the effects are illustrated in Figure 7.

## 4. Discussion

Several challenges may have obscured reliable group-level distinctions in earlier research on MLR characteristics in children with APD. Studies included disparate assessment protocols and diagnostic definitions that resulted in heterogenous participant groups, making it difficult to reliably compare group-level distinctions across studies. We sought to overcome this limitation by initially classifying participants into three well-defined dichotic listening profiles based on strict criteria for identification. By aligning classification with matched behavioral performance on two tests of dichotic listening skills, our approach allowed for a more precise evaluation of whether specific deficits in dichotic listening, rather than general weaknesses in auditory processing, can be associated with distinct patterns in the MLR. We chose to examine the MLR for ear- and electrode-related asymmetries because interaural asymmetry is a defining feature of amblyaudia, one common subtype of dichotic listening deficit [20,21]. In contrast, dichotic dysaudia, another prevalent dichotic deficit, is characterized by bilaterally reduced performance and is not associated with pronounced ear asymmetry [3].

The results showed that MLR outcomes aligned with the expected deficit profiles with respect to asymmetry in children with a typical REA during dichotic testing. As anticipated, the AMB group demonstrated the greatest interaural asymmetry, with one ear consistently outperforming the other. In contrast, the TYP group exhibited minimal asymmetry, with ear performance at age-appropriate levels, while the DD group displayed a similar minimal asymmetry with poor performance in both ears.

### 4.1. MLR Peak-to-Peak Amplitude: Symmetry vs. Asymmetry

#### 4.1.1. Ear Effect on Peak-to-Peak Amplitude

The larger responses in the left ear during the MLR can be understood in light of hemispheric specialization and stimulus characteristics. In dichotic listening tasks with verbal material, a REA is typically observed because right-ear input projects contralaterally to the left hemisphere [42], which is dominant for speech and linguistic processing [41]. Because contralateral auditory pathways are heavily myelinated [42] and highly efficient, they rapidly transmit verbal signals from the right ear to the language-dominant left hemisphere and in contrast, from the left ear to the right hemisphere for non-linguistic signals [41]. This interplay between stimulus type and hemispheric specialization helps to explain the larger MLR for left-ear click stimulus in individuals who would demonstrate higher performance during dichotic listening for right-ear input. Children with typical dichotic listening performance demonstrated a REA, whereas children with amblyaudia showed a more pronounced REA, consistent with their reduced behavioral performance for left-ear input during dichotic listening tasks.

Ear effects are useful indicators of interaural response symmetry. They are often attributed to subcortical disruptions, particularly at the level of the brainstem or thalamic relay nuclei, where contralateral pathways dominate auditory transmission [34,35]. The fact that a significant ear effect was found in the AMB group suggests that it may reflect a reliable neural correlate of the interaural imbalance seen in behavioral dichotic performance in these children. Importantly, while ear effects have frequently been reported in patients with structural subcortical lesions [34,35], the present findings demonstrate that such asymmetries can also emerge in the absence of documented structural damage, reflecting instead functional alterations in auditory processing.

In an MLR study that examined inter-trial phase coherence of cortical neural oscillations, children with AMB produced significantly stronger right ear phase-locked oscillatory responses, especially for inputs to the right ear despite demonstrating the normal larger amplitude of the evoked response from the left ear [20]. The lowest magnitude of oscillatory responses in children with AMB occurred for input to the left ear ascending to the right hemisphere along the presumptive dominant pathway. This result suggests reduced trial-to-trial variability, leading to stronger neural synchronization and phase-locking in the right ear neural pathway in children with AMB that was not reflected in the simpler right versus left amplitude measure of the MLR response. Because these stronger oscillatory responses are observed following input to the right ear which produces a smaller amplitude measure than the left ear in children with AMB, it seems likely that these two electrophysiologic measures are not directly related. Evaluating inter-trial phase coherence of cortical neural oscillations in children with DD may assist with understanding this apparent discrepancy in ear-level findings.

Another study examined responses across multiple electrode sites but only in adults. Malavolta et al. investigated Na and Pa amplitudes and latencies at C3 and C4 in response to left and right ear stimulation. Their results showed that young adults with altered auditory skills exhibited significantly smaller Na and Pa amplitudes at C3 for both ears compared to controls, whereas no group differences were observed at C4 [43]. The authors attributed these findings to impaired auditory decoding within the left hemisphere, where C3 electrode site reflects input to regions including Wernicke’s area, a critical site for the comprehension of auditory information. They related this finding of weaker responses over the left hemisphere to decoding deficits in these participants that may degrade acoustic signals and contribute to listening difficulties.

Group-level differences were also reported by Abdollahi et al. and Schochat et al., both of whom found significantly reduced MLR amplitudes in children with APD compared to controls [18,37]. Our findings diverge from these in that we did not observe overall amplitude reductions distinguishing dichotic listening profiles from typical controls. One likely reason our findings differ from prior studies is the way participants were categorized. Whereas Schochat et al. and Abdollahi et al. compared broad APD and control groups, we classified children into more specific subtypes based on their dichotic listening profiles. This finer-grained approach revealed patterns in the MLR that closely matched behavioral expectations rather than broad group-level amplitude reductions.

However, our within-group analyses revealed consistent asymmetries, particularly an ear effect at C4 with stronger responses to left-ear input. Notably, Schochat et al.’s descriptive data also showed this pattern, with left-ear stimulation producing larger amplitudes than right-ear stimulation in children with APD, although it was not explicitly highlighted.

Finally, Mattsson et al. reported a left-ear advantage in MLR amplitude, with larger responses for left-ear stimulation. Our results are consistent with this observation and extend it by localizing the effect specifically to electrode site C4. Although Mattsson did not include lateralized electrode sites such as C3 and C4, their finding at Fz still reflects the dominance of left-ear neural responses to non-verbal stimuli, which aligns with our observation of larger PP amplitudes for left-ear input [19].

#### 4.1.2. Electrode Effect

In the present study, analyses of the electrode effect revealed no significant differences in PP amplitude between C3 and C4 within any of the dichotic listening profiles. This suggests that, at the group level, MLR responses were statistically similar across hemispheric recording sites, consistent with the expectation of symmetry in the TYP group and plausibly explained in the DD group by their bilaterally poor but balanced performance on dichotic listening tasks. In contrast, asymmetries might have been anticipated in the AMB group, given prior evidence of hemispheric imbalance. For example, time-frequency analyses of the MLR have shown enhanced right-hemisphere activation in children with AMB [20], while fMRI studies using dichotic word stimuli have demonstrated increased left-hemisphere activation [21]. The inconsistency regarding the dominant hemisphere may reflect an interaction between stimulus type and hemispheric specialization [38,41,42]. While prior studies reporting hemispheric asymmetries might suggest the presence of an electrode effect, we did not observe such an effect. One possible explanation is that the lack of a significant electrode effect in the AMB group indicates that conventional peak-based measures may be insufficiently sensitive to detect more nuanced hemispheric differences, which are more readily revealed through time-frequency or neuroimaging approaches.

### 4.2. MLR Peak-to-Peak Latency

#### 4.2.1. Group-Level Comparisons

Our analysis of PP latencies revealed further nuances in auditory processing across groups. Latency patterns were modulated by group, stimulus rate, and ear of stimulation, highlighting the complex interplay of these factors. To disentangle these interaction effects, we conducted detailed within-group analyses, examining how ear of stimulation, and stimulus rate influenced MLR latency at each electrode site. This in-depth approach provided valuable insights into the neural mechanisms and revealed different electrophysiological patterns in children with AMB and DD.

Prolonged latencies in the DD group were most evident under fast-rate stimulation of the right ear. At the group level, this effect appeared at electrode C3 where children with DD showed significantly longer latencies compared to other groups, suggesting increased neural processing demands under rapid input conditions and highlights temporal vulnerabilities that affect right-ear inputs. Such delays may underlie the poor right-ear performance in dichotic listening typically seen in children with DD, indicating reduced temporal efficiency in the right-ear auditory pathway under fast stimulation rates. This deterioration could impact performance across both verbal and non-verbal stimuli in challenging listening environments. While no prior electrophysiological studies have specifically examined MLR responses in children with DD, it is possible to tentatively interpret these findings by extrapolating from mechanisms proposed in earlier research comparing results in children with AMB to age-appropriate controls. In children with AMB, right-ear stimulation was associated with stronger phase-locking, facilitating faster neural transmission along the right-ear pathway [38]. By contrast, the prolonged latencies observed in the DD group for right-ear stimulation in this study may suggest a relative weakness in phase synchrony along the right-ear auditory pathway. However, this remains speculative in the absence of direct phase-locking data for the DD population. Further research is necessary to investigate whether such phase-locking asymmetries exist in DD and to clarify the neural underpinnings of the observed latency delays.

#### 4.2.2. Ear Effect on Peak-to-Peak Latency

In contrast to the present findings, several previous studies did not report significant latency shifts in children with APD compared to controls. For example, Malavolta et al. (2022), Schochat et al. (2010), and Mattsson et al. (2019) all examined MLR responses and found no group-level latency differences [18,19,43]. A likely explanation for this discrepancy lies in the stimulation rate. In each of these studies, click stimuli were presented at a relatively slow rate, whereas all latency shifts observed in the current study emerged under fast-rate stimulation. This suggests that slow stimulation rates may be insufficient to reveal the increased temporal precision demands that emerge when we evaluate dichotic listening skills in children. An exception is Abdollahi et al. (2019), who did report longer Na and Pa latencies in the APD group despite using slow stimulation [37]. However, their larger sample size may have provided the statistical power necessary to detect effects that were absent in smaller cohorts, potentially accounting for the divergence from other reports.

Importantly, when the analysis was restricted to the DD group alone, a within-group ear effect also emerged at electrode C4, with right-ear stimulation producing longer PP latencies than left-ear stimulation. Taken together with the previous between-group finding at electrode C3, these results show a consistent pattern. The contralateral effect observed between groups (right-ear input yielding longer PP latency at C3) and the ipsilateral effect observed within the DD group (right-ear input yielding longer PP latency at C4) both point to the same conclusion that both ipsilateral and contralateral pathways reveal a weakness in sustaining efficient temporal processing for right-ear inputs under fast stimulation. This finding is also consistent with dichotic listening performance, since the DD group is the only group that demonstrates a right-ear weakness during dichotic listening tasks.

### 4.3. MLR Stimulus Rate Sensitivity

The DD group exhibited distinct patterns of rate sensitivity, highlighting additional weaknesses in temporal auditory processing. Under fast-rate stimulation, PP latencies were significantly prolonged at both C3 and C4, suggesting that rapid input places heightened processing demands on right-ear pathways. Taken together with the two preceding findings, this pattern may hypothetically reflect a weaker phase-locking mechanism within right-ear pathways in the DD group. Such reduced neural synchrony would render the right ear less capable of maintaining temporal precision, thereby increasing processing demands under conditions of rapid stimulation. Future research is needed to validate these possibilities.

Notably, the lack of significant rate sensitivity in children with AMB aligns with prior electrophysiological findings by Momtaz et al., who reported that AMB responses were largely resistant to stimulus rate manipulations compared to age-matched peers [44]. This resistance was evident across both ears and at cortical sites in both hemispheres, suggesting a fundamental rigidity in auditory temporal encoding. The observed rate insensitivity was interpreted as a form of “rigid tagging,” wherein external auditory events are encoded in a temporally inflexible manner, potentially limiting adaptive modulation of neural timing. The present findings corroborate this pattern, providing additional evidence that the auditory system in AMB may operate with reduced temporal plasticity, a characteristic that distinguishes it from other forms of auditory dysfunction, such as DD.

Rate-dependent latency effects are well documented in earlier auditory evoked potentials, particularly the auditory brainstem response (ABR), where increasing stimulus repetition rate reliably produces prolonged peak latencies. Studies of click-evoked ABRs have shown that faster stimulation rates result in delayed wave V latency, reflecting neural refractory limitations and reduced temporal recovery within the auditory pathway, whereas slower rates yield shorter latencies and clearer waveform morphology [45].

The presence of similar rate-related latency prolongation in the present MLR findings suggests continuity in rate sensitivity across multiple levels of the auditory system, extending from the brainstem to thalamocortical processing stages. Clinically, comparisons of latency behavior across stimulus rates have been used in ABR testing to probe neural synchrony and temporal integrity, and the current findings indicate that stimulus rate manipulation in MLR recordings may provide complementary information for identifying auditory processing inefficiencies, particularly in children with dichotic dysaudia.

### 4.4. Summary of Findings and Clinical Implications

The present study demonstrates that MLR characteristics closely parallel distinct behavioral profiles of dichotic listening performance in children. Specifically, children withAMB exhibited pronounced ear-related amplitude asymmetries in the MLR, particularly larger responses to left-ear stimulation at electrode site C4, consistent with their hallmark behavioral interaural asymmetry. In contrast, children with DD showed relatively symmetric MLR amplitudes but displayed rate-dependent prolongation of peak-to-peak latency for right-ear stimulation at faster presentation rates, suggesting a vulnerability in temporal auditory processing rather than asymmetric neural activation. Typically performing children demonstrated small, expected asymmetries without significant latency delays. Together, these findings indicate that MLR measures provide objective electrophysiological markers that differentiate subtypes of dichotic listening deficits, supporting their potential clinical utility for refining diagnosis beyond behavioral testing alone. Future studies should extend this work by incorporating binaural stimulation paradigms, and longitudinal designs examining MLR changes before and after auditory training, which may further elucidate neural mechanisms underlying intervention-related plasticity and enhance clinical applicability.

### 4.5. Limitations

Several limitations should be considered when interpreting the findings of the present study. First, although participants were carefully classified into well-defined dichotic listening profiles based on consistent performance across standardized behavioral measures, the overall sample size within each group, particularly the DD group, was modest. While this limitation is common in electrophysiological studies involving pediatric clinical populations, smaller group sizes may have reduced statistical power for detecting more subtle effects, particularly higher-order interactions involving electrode site, ear, and stimulus rate. As such, some non-significant trends observed in the data should be interpreted cautiously and warrant replication in larger cohorts.

Second, the study focused exclusively on children demonstrating a REA during dichotic listening, excluding a small subset of participants with a LEA. This decision was necessary to preserve interpretability of ear-related effects in the electrophysiological data; however, it limits the generalizability of the findings to children with atypical lateralization patterns. Future studies should specifically examine whether children with LEA exhibit systematic reversals or distinct MLR patterns that parallel their behavioral asymmetry. Handedness information was not available for all participants. Among participants for whom handedness data were available, the majority were right-handed, with four individuals showing no clear hand preference and two identified as left-handed.

Third, although relative PP amplitude and latency measures were intentionally used to reduce inter-subject variability, the auditory middle latency response is nonetheless characterized by substantial individual variability, particularly in pediatric populations undergoing ongoing neurodevelopment. While the use of within-subject comparisons and stratified analyses helped mitigate these effects, they cannot be eliminated.

Despite these limitations, the present study provides important evidence that carefully defined dichotic listening profiles are associated with distinct and interpretable patterns in MLR amplitude and latency, supporting the clinical and theoretical relevance of electrophysiological measures for characterizing subtypes of auditory processing difficulties in children

## 5. Conclusions

In summary, this study demonstrates that the auditory middle latency response reflects distinct neural processing patterns associated with AMB and DD. Asymmetries in PP amplitude and latency varied with distinct behavioral profiles and revealed sensitivity to ear and stimulus rate at different electrode sites. These findings underscore the importance of examining electrophysiologic responses in children grouped specifically based on consistent deficit patterns across two dichotic listening tests rather than diagnosing them with APD following a large, heterogeneous battery of tests that assess weakness across a potentially disparate variety of auditory processing skills. This study also highlights the potential utility of the MLR as an objective marker for characterizing weaknesses in the ascending auditory neural pathways in children identified with these very specific dichotic listening deficits.

## Figures and Tables

**Table 1:** Mean ± SE scores for dominant (right) and nondominant ears on the Randomized Dichotic Digits Test and Dichotic Words Test across dichotic listening profiles.

Groups	*n*	RDDT Scors (Mean ± SE)		DWT Scores (Mean ± SE)	
		Dominant (Right) Ear	Nondominant Ear	Dominant (Right) Ear	Nondominant Ear
TYP	11	98.15 ± 0.72	92.38 ± 2.16	93.33 ± 2.30	84.88 ± 2.81
AMB	17	87.30 ± 3.31	65.05 ± 4.22	86.66 ± 2.42	56.53 ± 5.40
DD	9	79.16 ± 3.45	72± 3.85	80.05 ± 4.61	74.66 ± 3.52

**Table 2:** Participant-level MLR PP amplitude means (µV), collapsed across stimulus rate and electrode site.

Participant ID	Group	Left Ear PP Amplitude (µV)	Right Ear PP Amplitude (µV)
P 1	AMB	0.74	0.65
P 2	AMB	0.45	0.54
P 3	AMB	0.60	0.16
P 4	TYP	1.01	1.13
P 5	TYP	1.14	1.18

**Table 3:** MLR PP Latency (Mean ± SE) by Stimulus Rate Within Each Dichotic Listening Profile.

Groups	*n*	Stimulus Rate	PP Latency (Mean ± SE)
TYP	11	Slow	13.91 ± 0.60
		Fast	13.81 ± 0.41
AMB	17	Slow	13.30 ± 0.37
		Fast	12.43 ± 0.39
DD	9	Slow	11.56 ± 0.67
		Fast	14.52 ± 0.68

**Table 4:** MLR PP latency (mean ± SE, ms) across ears and stimulus rates for all participants (*n* = 37), collapsed across electrode sites.

Ear	Stimulus Rate	PP Latency (Mean ± SE)
Right	Slow	12.70 ± 0.43
	Fast	13.60 ± 0.39
Left	Slow	13.52 ± 0.40
	Fast	12.81 ± 0.39

**Table 5:** PP Latency (Mean ± SE) by Stimulus Rate at Electrode Sites C3 and C4 in the DD Group (*n* = 9).

Electrode	Stimulus Rate	PP Latency (Mean ± SE)
C3	Slow	10.74 ± 1.19
	Fast	16.04 ± 1.03
C4	Slow	12.38 ± 1.25
	Fast	16.31 ± 1.02

## Data Availability

The original contributions presented in this study are included in the article. material. Further inquiries can be directed to the corresponding author.
